# Development, optimization, and evaluation of PEGylated brucine-loaded PLGA nanoparticles

**DOI:** 10.1080/10717544.2020.1797237

**Published:** 2020-07-30

**Authors:** Heba S. Elsewedy, Bandar E. Al Dhubiab, Mahmoud A. Mahdy, Hanan M. Elnahas

**Affiliations:** a Department of Pharmaceutics and Industrial Pharmacy, Faculty of Pharmacy, Zagazig University, Zagazig, Egypt; b Department of Pharmaceutical Sciences, College of Clinical Pharmacy, King Faisal University, Saudi Arabia

**Keywords:** Nanoparticle, optimization, PLGA, PEG, Brucine

## Abstract

The application of nanotechnology to drug delivery systems for cancer therapy has progressively received great attention. The most heavily investigated approach is the development of nanoparticles (NPs) utilizing biodegradable and biocompatible polymers such as poly (lactic-*co*-glycolic acid) (PLGA). These NPs could be further improved by surface modification utilizing a hydrophilic biodegradable polymer such as polyethylene glycol (PEG) to achieve passive targeting. Modified NPs can deliver drugs such as brucine (BRU), which has shown its potential in cancer therapy. The objective of the current investigation was to develop and evaluate the passive targeting of long-circulating PLGA NPs loaded with BRU. NPs were characterized in terms of drug-excipient compatibility studies, including FTIR and DSC; physicochemical evaluations including particle size, zeta potential, morphological evaluation, entrapment efficiency and percentage yield; total serum protein adsorbed onto NP surfaces; and *in vitro* release of the loaded drug. Factorial design was employed to attain optimal PLGA-loaded NPs. Finally, the *in vivo* anti-tumor activity of BRU-loaded PLGA NPs was evaluated in tumor-bearing mice. The NPs obtained had smooth surfaces with particle sizes ranged from 94 ± 3.05 to 253 ± 8.7 nm with slightly positive surface charge ranged from 1.09 ± 0.15 to 3.71 ± 0.44 mV. Entrapment of BRU ranged between 37.5 ± 1.8% and 77 ± 1.3% with yields not less than 70.8%. Total protein adsorbed was less than 25.5 µg total protein/1 mg NP. *In vitro* drug release was less than 99.1% at 168 h. Finally, significant reductions in tumor growth rate and mortality rate were observed for PEG PLGA NP formulations compared to both BRU solution and naked NPs.

## 1. Introduction

Cancer is a widespread disease in which cells grow and divide abnormally and out of control, resulting in a mass known as a tumor. There is considerable interest in new technologies that can differentiate between normal and cancer cells and specifically target the tumor. The most remarkable approach is targeted drug delivery (TDD), in which the drug is incorporated into a nanocarrier such as a liposome, niosome, nanoemulsion, or nanoparticle. TDD increases both drug efficacy and reduces drug toxicity, and it could overcome a wide range of obstacles such as drug solubility and instability, as well as facilitate drug delivery to the target cell. Different drug targeting strategies exist, namely passive and active targeting (Mohamed et al., 2019). Passive targeting depends on a unique phenomenon of most solid tumors known as the enhanced permeability and retention (EPR) effect, in which molecules of certain sizes are preferentially taken up by and accumulate in the tumors (Danaei et al., 2018). However, intravenously administered nanocarriers loaded with anticancer drugs are normally taken rapidly out of blood circulation by the reticuloendothelial system (RES). To prolong the circulation time of these carriers, and, therefore, their targeting to tumor tissue, a hydrophilic polymer (polyethylene glycol, PEG) layer is introduced on the surface of the nanocarriers (Shehata et al., 2016). Such modification would prevent the adsorption of plasma proteins (opsonin), which has a major role in enhancing phagocytosis, and therefore extend the blood circulation time (Jörg et al., 2007).

NPs are considered to be a drug delivery system that enables unique approaches for cancer treatment, and to be one of the most important means utilized in nanomedicine (Jiang et al., 2007). A large number of NP delivery systems have been developed, in which the drug to be delivered is dissolved, entrapped, and encapsulated within the matrix (Lövestam et al., 2010). NPs conjugated with biodegradable polymers such as PLGA have pulled considerable attention as a result of their ability for active and passive tumor‐targeting (Xiaowei et al., 2015). The external dimensions of NPs range from a few nanometers up to 1000 nm. It is well known that NPs coated with PEG can accumulate in different types of solid tumors due to the EPR effect; they are considered suitable vehicles for hydrophobic drugs, able to attain efficient tumor targeting with the fewest adverse reactions (Venkatasubbu et al., 2013; Siqi et al., 2019). Several methods were applied for NPs development including nanoprecipitation method (Peng et al., 2018), solvent evaporation method (Catarina et al., 2006), dialysis (Rao & Geckeler, 2011), and salting out (Sovan et al., 2011). Brucine (BRU) is a white, odorless, crystalline, and poorly water-soluble anticancer drug extracted from Strychnos nux-vomica seeds (Gupta & Chaphalkar, 2015). BRU is considered as a promising anticancer agent; it has antitumor activity, antiangiogenic effects, and anti-proliferative activity, therefore, can have anti-carcinogenic effects in different types of cancer (Shu & Xi-Peng, 2017). The current investigation is an attempt to incorporate BRU into PEG poly (lactic-*co*-glycolic acid) (PLGA) NPs to achieve passive targeting after intravenous administration. The BRU-loaded PLGA NPs were evaluated for physicochemical properties, drug-excipient compatibility, and *in vitro* drug release. The adsorption of serum proteins onto the surface of PLGA NPs was also quantified. Finally, the *in vivo* effect of BRU-loaded PLGA NPs on tumor volume and survival time was evaluated in MDA-MB-231 tumor-bearing mice.

## 2. Materials and methods

### 2.1. Materials

BRU was obtained from Alpha Chemika, (Mumbai, India). PLGA (50:50, MW 75,000), polyvinyl alcohol (PVA), and dichloromethane were purchased from Sigma Aldrich (St Louis, MO). Poly ethylene glycol-distearoylphosphatidyl ethanolamine (PEG-DSPE) was purchased from Lipoid LLC (Newark, NJ). Dulbecco’s modified Eagle’s medium (DMEM) and fetal bovine serum (FBS) were obtained from Sigma Aldrich (St. Louis, MO). Total protein colorimetric kits purchased from United Diagnostics Industry (Dammam, KSA) All other reagents were of the finest grade available.

### 2.2. Development of BRU-loaded PLGA NPs

#### 2.2.1. Development of BRU-loaded naked NPs (NNPs)

NNP formulations of BRU (Table 1) were prepared by a modified solvent evaporation method (Hoa et al., 2012). Required quantities of ingredients were weighed. BRU was dissolved in 5 ml dichloromethane, followed by adding PLGA, mixed well to dissolve completely and forming the organic phase. This organic phase was added drop-wise into the aqueous phase, containing PVA as surfactant, using glass syringe while homogenization at an optimized speed using a high-speed homogenizer (Polytron PT 3000, Kinematika, Switzerland). The homogenization was applied for about 5 min at 10,000 rpm and for 5 min at 15,000 rpm. The coarse emulsion was sonicated for about 2 min using probe sonicator to get the desired particle size. The resulting nanosuspension was then stirred for 2 h to evaporate the organic solvent (Govender et al., 1999). The NNPs were obtained after consecutive centrifugation using Amicon® ultra- 4 (Ultracel-10 K) at 6000 rpm for 30 min and washing with distilled water, which is repeated twice to remove the non-incorporated drug. The retained NNPs is re-suspended with 2 ml of distilled water and freeze-dried (Pedram & Azita, 2017).

**Table 1.** Composition of the prepared BRU-loaded PLGA NPs.

Batch no.	BRU (mg)	Dichloro-methane (ml)	PLGA (mg)	PEG-DSPE (mg)	PVA (mg)	Dist. water (ml)
NNP1	25	5	50	0	10	10
NNP2	25	5	75	0	10	10
NNP3	25	5	100	0	10	10
NNP4	25	5	50	0	20	10
NNP5	25	5	75	0	20	10
NNP6	25	5	100	0	20	10
NNP7	25	5	50	0	30	10
NNP8	25	5	75	0	30	10
NNP9	25	5	100	0	30	10
NP1	25	5	50	50	10	10
NP2	25	5	75	50	10	10
NP3	25	5	100	50	10	10
NP4	25	5	50	50	20	10
NP5	25	5	75	50	20	10
NP6	25	5	100	50	20	10
NP7	25	5	50	50	30	10
NP8	25	5	75	50	30	10
NP9	25	5	100	50	30	10

#### 2.2.2. Development of BRU-loaded PEG NPs

BRU-loaded PEG NP formulations (Table 1) were prepared by a modified solvent evaporation method (Hoa et al., 2012). As previously mentioned in NNPs preparation, required quantities of ingredients were weighed. BRU was dissolved in 5 ml dichloromethane, and then PLGA was added followed by PEG and mixed well to dissolve completely and forming the organic phase. The same procedure of developing NNPs was followed to obtain freeze dried PEG NPs.

### 2.3. Evaluation of formulation variables

In an attempt to improve drug targeting, NPs with surface modification using PEG were developed along with naked ones. For optimizing the concentration of aqueous solution, NPs were prepared using different concentrations of the surfactant. The surfactant concentration in the aqueous phase was 0.1, 0.2, or 0.3% PVA while keeping other parameters constant (Keum et al., 2011). Regarding the effect of polymer concentration on the entrapment of BRU, PLGA was used and studied in three different concentrations 50, 75, and 100 mg (Navneet et al., 2016).

### 2.4. Determination of drug-excipient compatibility studies

#### 2.4.1. Fourier transform infrared (FTIR) characterization

Drug excipient interaction was studied by FTIR spectroscopy (FTIR spectrophotometer, Shimadzu, Iraffinity-1S, Japan) by KBr pellet method. For the NP sample preparation, 5 μg of NPs was placed on the KBr plate and dried in vacuum. The FTIR spectra of all samples were recorded between 4000 and 400 cm^−1^. In this study, the spectra obtained for BRU, pure PLGA, pure PEG, PVA alone, and the prepared BRU NPs were analyzed by FTIR.

#### 2.4.2. Differential scanning calorimetry (DSC) characterization

To determine the physical state of BRU in the formulated NPs, DSC experiments were carried out for pure BRU, pure polymer and pure PLGA to identify the melting point peak. Subsequently, NPs loading with the drug were analyzed (Rubiana et al., 2006). The thermal analysis of the samples was determined using a DSC apparatus (DSC-60 Instrument, Shimadzu, Tokyo, Japan). The samples were heated at a rate of 10 °C/ min from room temperature to 350 °C with nitrogen atmosphere (Issa et al., 2013).

### 2.5. Characterization of BRU-loaded PLGA NPs formulations

#### 2.5.1. Particle size analysis and zeta potential

Particle size distribution, polydispersity indexes (PDI) and zeta potential of BRU-loaded NPs were measured using a Zetasizer apparatus (Malvern Instruments Ltd., Worcestershire, UK) at room temperature. The particle distribution was evaluated by measuring the dynamic light scattering of NPs. Zeta potential was assessed by determining the electrophoretic mobility (Shah et al., 2019).

#### 2.5.2. Morphological evaluation

The morphology of the prepared NPs was assessed by performing a scanning electron microscopy (SEM), JSM-6390LA, JEOL (Tokyo, Japan). NPs surface morphology was studied at different magnifications (1000–95,000). NPs were coated with gold under vacuum on metal stubs, and then examined at 15kv.

#### 2.5.3. Entrapment efficiency (EE %) and % yield of NPs

The entrapment efficiency of the formulated NPs was taken as the amount of BRU carried by the NPs. Initially, BRU acetonitrile solution (0.01 mg/mL) was prepared as a control solution. NPs equivalent to 5 mg of the drug were used for calculating the EE. The amount of drug entrapped was estimated by dissolving the NPs in 5 ml of acetonitrile and then apply sonication at 50 W for 5 min for fully extracting the drug and vortex at 1600 rpm for 15 min. Thereafter, centrifugation was applied at 3000 rpm for 15 min and the supernatant was collected, BRU concentration was determined at ƛ_max_ of 264 nm (Qin et al., 2012). Regarding the % yield, the developed NPs were collected and weighed carefully. % yield was calculated according to the following formula (Keum et al., 2011):

%yield=(weight of NP obtained/weight of drug and polymer)×100

### 2.6. Quantitative determination of serum protein adsorption onto NPs surface

Regarding the protein adsorption, NP preparation was suspended in 1 ml phosphate buffer saline (PBS) and incubated with same volume of fresh rat serum for 30 min at 37 °C ± 0.5. Then, the dispersion was separated from bulk serum proteins by centrifugation using Amicon® ultra- 4 (Ultracel-10 K) at 6000 rpm for 30 min (Sempf et al., 2013). NPs were collected and the amount of protein adsorbed on their surface was quantitatively assessed by total protein colorimetric kits (United Diagnostics Industry, Dammam, KSA).

### 2.7. In vitro drug release study of BRU from NPs

The *in vitro* release of BRU from BRU-loaded NPs was performed using dialysis bag diffusion technique (Morsy et al., 2019). The formulated NPs were kept in dialysis bags (12,000–14,000 DM-27, Millipore, Burlington, MA) immersed in 50 ml of PBS pH 7.4 at 37 °C using continuous magnetic stirring at 50 rpm. Samples of 1 ml were withdrawn from the receptor compartment at predetermined time intervals (0.5, 1, 2, 3, 6, 12, 24, 36, 48, 72, 96, 120, 144, and 168 h) and replaced by the same volume of fresh medium. Dissolution tests were performed in triplicate. The amount of BRU released was determined spectrophotometrically at ƛ_max_ of 264 nm (Mohammed & Urszula, 2014).

### 2.8. Experimental design study

Various trials were investigated, prior to establish the present optimization study by selecting various parameters like varied concentration of PLGA, PVA, rate of stirring and stirring time, the ratio of organic to aqueous phase, etc. Lastly, two independent factors were selected based on result obtained and their influence on the Brucine-PLGA NPs was studied. Three-level and two-factor factorial design experiment was developed using Design-Expert version 11.0 software (Stat-Ease, Minneapolis, MN). The selected critical variables are follows: Concentration of PLGA (X1) and Concentration of PVA (X2). The independent variables were taken at three different levels (–1, 0, 1) as shown in Table 2, where the particle size in nm (Y1), % yield (Y2), and the protein absorbed in µg/mg (Y3) were considered as dependent variables. The design matrix was produced by software consisted of 09 experiments shown in Table 3, all the experiments were performed in a random order to reduce the effect of bias and unknown variables in the obtained results. All other parameters (temperature, rate of stirring and time, the ratio organic to aqueous phase and evaporation time) were kept as constant to minimize instability (Kozaki et al., 2017; Ismail et al., 2019). 2D Contour plot and 3D-response surface plot were created for illustrative representation of the volume of the response. Statistical analysis of generated data was performed by ANOVA provided by the software. A mathematical modeling was carried out by using following equation to obtain a first-order polynomial equation depending on significant influences among two factors (*X*_1_ and *X*_2_) of the factorial design model:

$$Y=b_{o}+b_{1}X_{1}+b_{2}X_{2}+b_{12}X_{1}X_{2}+b_{11}X_{1}{}^{2}+b_{22}X_{2}{}^{2}$$

**Table 2.** Selected critical independent variable and their level of variation.

Independent variable	Symbol	Level of variation		
		−1	0	+1
Conc. of PLGA (mg)	X1	85	100	115
Conc. of PVA (mg)	X2	15	20	25

**Table 3.** Software generated design matrix.

Experiment number	Formulation code	Conc. of PLGA (X1)	Conc. of PVA (X2)
1	NP01	85	15
2	NP02	100	15
3	NP03	115	15
4	NP04	85	20
5	NP05	100	20
6	NP06	115	20
7	NP07	85	25
8	NP08	100	25
9	NP09	115	25

where *Y* is the dependent variable, while *b*_0_ is the intercept, *b*_1_, *b*_2_, *b*_12_, *b*_11_, and *b*_22_  are the regression coefficients; *X*_1_, and *X*_2_ are the main factors; *X*_1_*X*_2_ are the interactions between main factors, and X1^2^ and X_2_^2^ are the polynomial terms.

### 2.9. Cell line

MDA-MB-231 cancer cells were purchased from the American Type Culture Collection (ATCC; Manassas, VA) through college of science, King Faisal University, KSA. Male Balb/c mice of 8–10 weeks were obtained from animal breeding center, college of science, King Faisal University. MDA cells were cultured in DMEM, supplemented with 100 U/ml penicillin, 100 μg/ml streptomycin, 20 μg/ml gentamicin, and 10% heat-inactivated FBS at 37 °C under 5% CO_2_/95% air (Yuan et al., 2018).

### 2.10. Animal model

To prepare tumor-bearing mice, 5 million tumor cells were subcutaneously inoculated into the right back of mice (Yuan et al., 2018). The animals were checked three times a week at the site of injection for the tumor development.

### 2.11. In vivo anti-tumor activity evaluation of BRU-loaded PLGA NPs in MDA tumor-bearing mice

This investigation was designed to evaluate the *in vivo* antitumor activity of optimized BRU-loaded PLGA NPs on MDA tumor bearing mice. After growing up of tumor volume to approximately 150 mm^3^ after inoculation of MDA cells, 20 tumor-bearing mice were randomly divided into 4 groups, 5 mice per group, as follows:

- Group 1: Considered as control and administered saline.
- Group 2: Received BRU solution (2 mg/kg).
- Group 3: Treated with NPs formulation namely NP6 containing equivalent amount of drug (2 mg/kg).
- Group 4: Treated with NPs formulation namely NNP6 containing equivalent amount of drug (2 mg/kg).

The selected BRU NPs, prepared with 0.2% PVA, were administered intravenously through the tail vein at a dose of 2 mg/kg. Drinking, diet, and movement of all tumor-bearing mice were observed and weighed daily during the treatment. The antitumor activity was estimated in terms of the tumor volume that was monitored every day and evaluated over 20 days. Tumor volume was measured with caliper in two dimensions, and calculated using the following equation:

Tumor volume (mm^3^) = longer diameter × (shorter one)^2^ × 0.52 (Lee et al., 2005). The experiment is terminated as one of the mice in either group died (Ogawara et al., 2008). The tumor growth rates for each NPs preparation was calculated from the slope of tumor volume-time curve. In addition, the survival time of tumor-bearing mice after the treatment was evaluated.

### 2.12. Statistics

All data were recorded as mean ± standard deviation. Data from treated groups were compared with data from the control group by applying a one-way analysis of variance (ANOVA) followed by the least significant difference (LSD) as a post-hoc test, using SPSS statistics software, version 9 (IBM Corporation, Armonk, NY). The level of *p* < .05 was considered statistically significant for all tests.

## 3. Results and discussion

### 3.1. Determination of drug-excipient compatibility studies

#### 3.1.1. FTIR characterization

**Figure 1.**
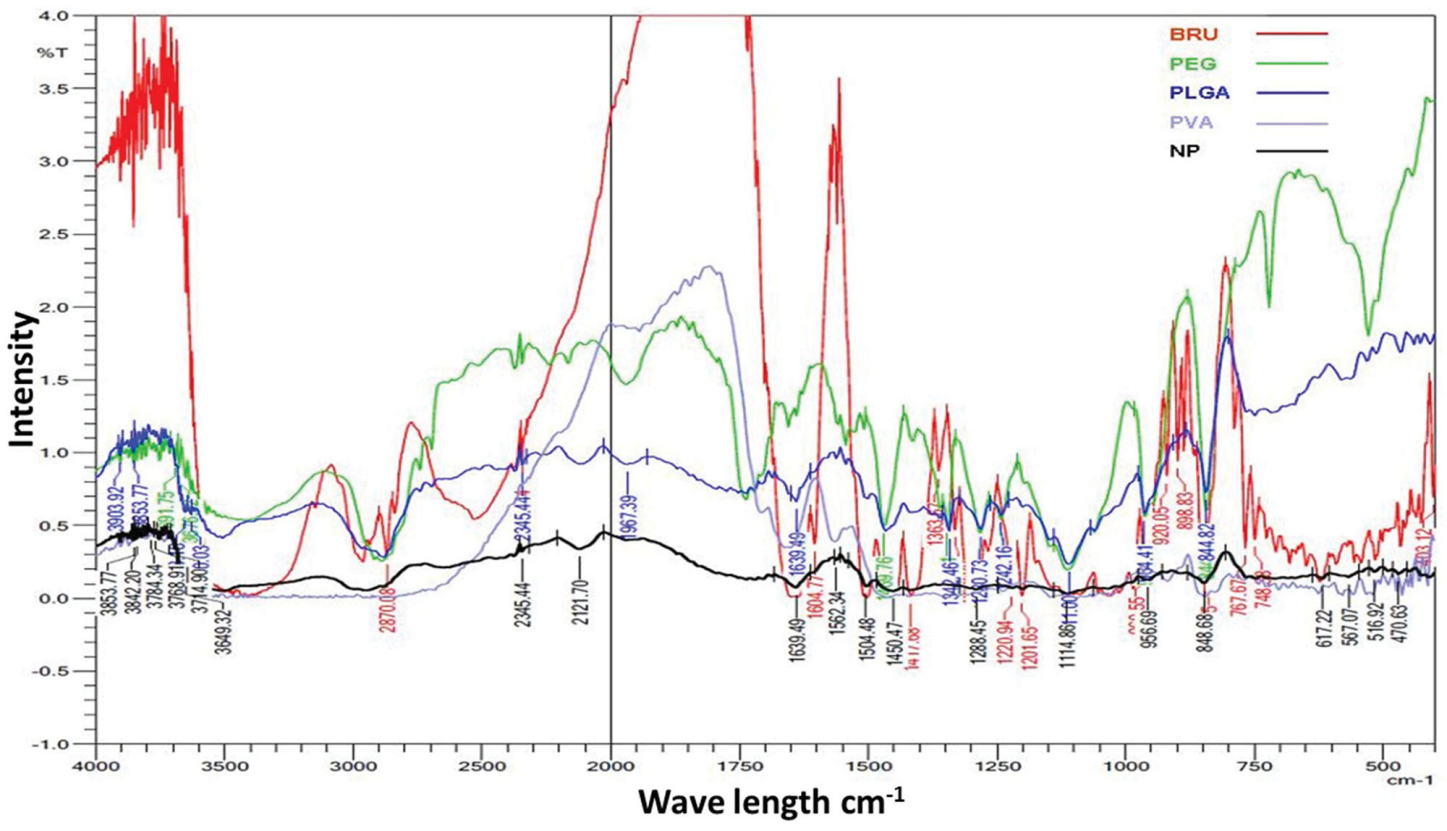
FTIR spectra of pure BRU, PEG, PLGA, PVA, and NP formulations prepared with PVA.

The possibility of non-covalent interactions between BRU and the polymers utilized in NPs manufacture was investigated by FTIR spectroscopy (Figure 1). Infrared spectra can provide detailed information about the structures of molecular compounds, allowing comparisons between pure compounds and mixtures. The spectrum for BRU showed a characteristic carbonyl –C = O stretch at 1653 cm^−1^, an aromatic stretch around 1500 cm^−1^, and peaks at 2842, 2868, 2903, and 2928 cm^−1^ that relate to the C–H bonds of saturated carbons; this spectrum confirmed the purity of the BRU. These results are in agreement with Zhipeng et al. (2013), whose work identified the same characteristic peaks for BRU. Pure PLGA sample showed peaks such as –CH, –CH2, –CH3 stretching (2850–3000 cm^−1^), carbonyl –C = O stretching (1700–1800 cm^−1^), C–O stretching (1050–1250 cm^−1^), and –OH stretching (3200–3500 cm^−1^), and all of these were broad. Absorption peaks of PVA are shown at about 3247.5 cm^−1^ for –OH stretching and at 1082 and 1414.5 cm^−1^ for the –C–O group (Rodríguez et al., 2007). In case of formulated BRU-NP, the sharp carbonyl stretch peak of the drug was very low and that indicate the non-covalent interaction, mostly the hydrogen bond with OH of the PEG. On the other hand, the finger print region of BRU was always present when mixing BRU with polymers indicating that the drug included in the formulation.

#### 3.1.2. DSC characterization

**Figure 2.**
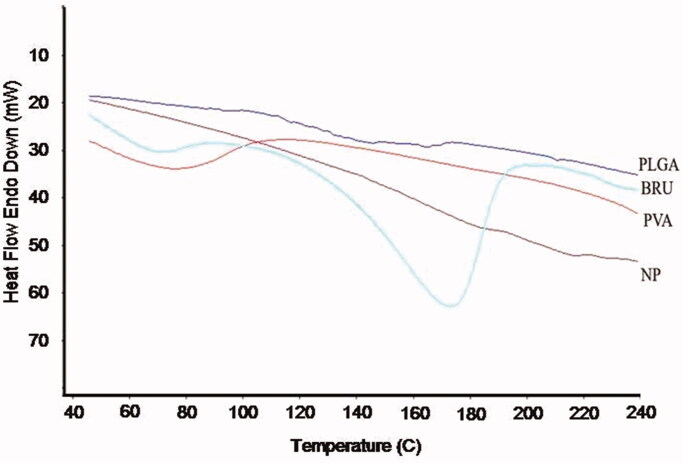
DSC thermogram of BRU, PLGA, PVA, and BRU-loaded PLGA NPs.

The potential for physical interaction between BRU and PLGA present in the NP formulation was evaluated by DSC (Figure 2). The thermogram of pure BRU had an endothermic peak at 178 °C corresponding to its melting point and decomposition, which indicates that the drug could be in a crystalline form. The absence of the sharp peak in the thermogram of loaded NPs could be an evidence that there was no crystalline drug in the NP formulation. This indicates that the crystal form of the drug has been reduced in the prepared NP and being in the amorphous form (Issa et al., 2013). In addition, no melting point was observed for pure PLGA polymer, confirming its amorphous nature.

### 3.2. Characterization of BRU-loaded PLGA NPs

#### 3.2.1. Particle size analysis and zeta potential

**Figure 3.**
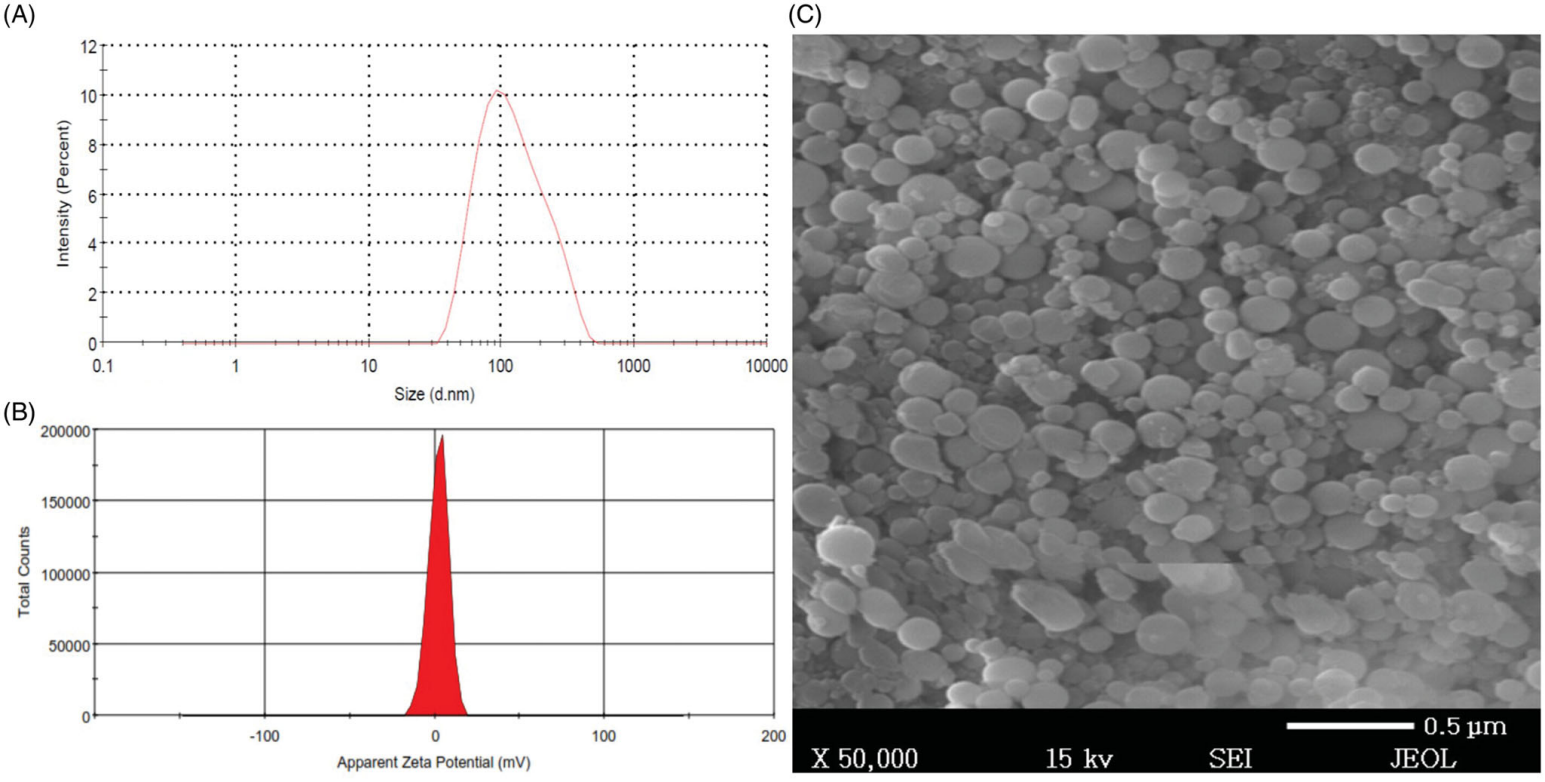
Size distribution, zeta potential, and scanning electron microscope of BRU-loaded PLGA NPs prepared with PVA. A: size distribution. B; zeta potential. C; scanning electron microscopic image.

The particle size and size distribution of BRU-loaded NP preparations were evaluated; results are reported in Table 4, and a representative sample is shown in Figure (3(A)). Particle sizes of NNPs ranged between 65 ± 4.0 and 206 ± 7.7 nm, with (PDI) of 0.41 and 0.297, respectively. However, the corresponding values for PEG NPs ranged between 94 ± 3.05 and 253 ± 8.7 nm, with PDIs of 0.34 and 0.368, respectively. The particle size of all PEG NPs were significantly different from their naked counterparts at *p* < .05. The obtained PDI indicates that the particle size distribution falls within a narrow range as stated previously by Ahmed et al. (2015). The increase in size of BRU-loaded NPs is ascribed to deposition of a polymeric coating (PEG) on the NP surface.

**Table 4.** The particle size, PDI, and zeta potential of prepared BRU-loaded PLGA NPs:.

Formulation	Particle size (nm ± SD)	PDI	Zeta potential (mV ± SD)	Formulation	Particle size (nm ± SD)	PDI	Zeta potential (mV ± SD)
NNP1	199 ± 3.0	0.337	−16.6 ± 1.7	NP1	215 ± 4.1	0.423	2.42 ± 0.37
NNP2	204 ± 8.1	0.542	−19.7 ± 3.0	NP2	238 ± 3.6	0.318	1.09 ± 0.15
NNP3	206 ± 7.7	0.297	−22.8 ± 2.3	NP3	253 ± 8.7	0.368	1.79 ± 0.83
NNP4	110 ± 2.08	0.211	−23.3 ± 5.2	NP4	124 ± 0.57	0.210	1.36 ± 0.12
NNP5	110 ± 1.0	0.192	−24.2 ± 5.5	NP5	134 ± 0.57	0.257	2.41 ± 0.11
NNP6	121 ± 0.0	0.380	−25.4 ± 2.1	NP6	161 ± 4.5	0.378	2.17 ± 0.57
NNP7	65 ± 4.0	0.410	−24.6 ± 4.5	NP7	94 ± 3.05	0.340	2.64 ± 0.11
NNP8	93 ± 2.8	0.320	−25.5 ± 2.0	NP8	113 ± 0.57	0.232	3.23 ± 0.62
NNP9	98 ± 5.03	0.450	−31.1 ± 7.2	NP9	115 ± 1.15	0.390	3.71 ± 0.44

Values are expressed as mean ± standard deviation (SD) *n* = 3 and were analyzed by Student’s *t*-test. All PEG NP formulations *p* < .05 compared to their naked counterpart.

Regarding the electrical charge of the surface, zeta potential is considered to be a significant parameter for the identification of NP surface charge and the stability of the formulation. Zeta potential of BRU-loaded NP preparations were evaluated and results are shown in (Table 4), a representative sample is shown in Figure 3(B). It was found that zeta potential of NNPs ranged between −16.6 ± 1.7 and −31.1 ± 7.2 mV, whereas, the corresponding values for PEG NPs ranged between 1.09 ± 0.15 and 3.71 ± 0.44 mV. It is obvious that NNPs tend to carry a characteristic negative charge which appears to be attributable to negatively charged carboxyl groups on PLGA surface (Wang et al., 2013). Certainly, surface modification of NNPs with PEG tends to change its surface charge to positive or neutral due to the displacement of ionic layer to further distance from the NP by the chain of PEG (Patel et al., 2012). This explain the considerable targeting of PEGylated NPs which is expected to be due to the electrostatic attraction between positive charge of PEGylated NPs and negative one of cancer cell surfaces (Yang et al., 2009).

#### 3.2.2. Effect of surfactant concentration on particle size

Surfactant concentration has been shown to have great influence on NP particle size. As shown in Table 4, using different BRU:PLGA ratio (1:2, 1:3, and 1:4) and PVA (0.1%, 0.2%, and 0.3%), the particle size ranged from 65 ± 4.0 to 206 ± 7.7 nm for NNP7 and NNP3, respectively. Since increasing in PVA concentration in all BRU:PLGA ratio resulted in decreasing particle size of NNPs. Similar results were obtained for PEG NPs where NP sizes likewise decreased with increasing PVA concentration as the size ranged between 94 ± 3.05 and 253 ± 8.7 nm for NP7 and NP3, respectively. A significant variation in particle sizes were obtained for different NNPs and PEG NPs with various surfactant concentrations (*p* < .05). The small particle size of prepared NPs could be attributed to the high concentration of surfactant, which would prevent the coalescence of globules, protect and stabilize droplets formed in the emulsion process, and result in smaller emulsion droplets (Rizwan et al., 2019).

#### 3.2.3. Morphological evaluation

NPs morphology was investigated using SEM; Figure 3(A) shows the size distribution of the selected BRU-loaded NPs namely NP6 and Figure 3(B) shows representative images of same preparation (NP6). The NPs possessed smooth surfaces and exhibited spherical shapes with separated particles or aggregation, which confirmed the suitability of the parameters selected for NP preparation. Our results are in accordance with the findings of Prakash et al. (2017), which confirmed the spherical shape and smooth surfaces of PLGA-encapsulated nattokinase polymeric NPs prepared with PVA.

#### 3.2.4. Percent yield of NPs

The percent yield of BRU-loaded NPs was determined (Table 5). For naked preparations, the percent yield ranged from 69.6 ± 0.6% to 92.9 ± 1.5%, while those of their PEG counterparts ranged from 70.8 ± 1.4% to 94.5 ± 1.1%. However, no significant difference was obtained for PEG NPs and their naked counterpart (*p* < .05). It could be inferred that increasing the concentration of PLGA polymer resulted in increased practical yield (Rekha et al., 2014).

**Table 5.** Entrapment efficiency and % yield of BRU-loaded PLGA NPs:.

Formulation	Entrapment efficiency % ± SD	Yield % ± SD	Formulation	Entrapment efficiency % ± SD	Yield % ± SD
NNP1	69.1 ± 2.1	87.8 ± 1.6	NP1	71.7 ± 1.4	89.3 ± 1.1
NNP2	70.6 ± 0.9	90.7 ± 3.7	NP2	73.2 ± 1.9	92.3 ± 1.8
NNP3	74.0 ± 2.5	92.9 ± 1.5	NP3	77 ± 1.3	94.5 ± 1.1
NNP4	49.9 ± 1.5	82.4 ± 3.4	NP4	52.5 ± 1.6	83.8 ± 1.5
NNP5	52.3 ± 2.4	83.2 ± 2.9	NP5	54.2 ± 1.1	84.6 ± 1.2
NNP6	59.6 ± 3.4	86.1 ± 2.6	NP6	58 ± 1.5	85.3 ± 1
NNP7	39.1 ± 1.9	69.6 ± 0.6	NP7	37.5 ± 1.8	70.8 ± 1.4
NNP8	40.1 ± 2.1	74.2 ± 2.9	NP8	39.1 ± 1.5	75.4 ± 1
NNP9	41.9 ± 1.4	80.0 ± 3.7	NP9	41.1 ± 1.1	79.3 ± 1.4

Values are expressed as mean ± standard deviation (SD), *n* = 3 and were analyzed by Student’s *t*-test, *p* < .05.

#### 3.2.5. Effect of surfactant on entrapment efficiency (EE) of BRU

Applying different concentrations of surfactant greatly influenced the EE of NPs (Table 5). With BRU: PLGA 1:2, increasing the concentration of PVA from 0.1% to 0.3% decreased the EE from 69.1 ± 2.1% to 39.1 ± 1.9% (NNP1 and NNP7, respectively). The same situation with BRU: PLGA 1:3 and 1:4 (Table 3), that saw a decrease in EE from 70.6 ± 0.9% to 40.1 ± 2.1% (NNP2 and NNP8) and from 74.0 ± 2.5% to 41.9 ± 1.4% (NNP3 and NNP9), respectively. The effect of surfactant concentration on EE was also evaluated for PEG NP preparations (Table 3). When using BRU:PLGA 1:2, increasing the concentration of PVA from 0.1% to 0.3% decreased EE from 71.7 ± 1.4% to 37.5 ± 1.8% (NP1 and NP7). Likewise, for BRU: PLGA 1:3 and 1:4, increasing PVA decreased EE from 73.2 ± 1.9% to 39.1 ± 1.5% (NP2 and NP8) and from 77 ± 1.3% to 41.1 ± 1.1% (NP3 and NP9), respectively. The drop in EE with increasing PVA could be attributed to greater release of the drug into the aqueous phase during mixing, leaving fewer drug molecules in the emulsion droplets to interact with PLGA molecules, resulting in decreased EE (Song et al., 2008). Based on our experimental data, using 0.2% PVA seems to be sufficient to prepare NPs with small particle size and appropriate EE.

#### 3.2.6. Effect of drug:polymer concentration on particle size

To study the effect of drug: polymer concentration on particle size, BRU-loaded naked PLGA and PEG-coated NPs were prepared with various concentrations of PLGA polymer (50 mg, 75 mg, and 100 mg). The surfactant concentration was kept constant in all formulations. Upon using 0.1% PVA and increasing drug:PLGA concentration from 1:2 to 1:4, the particle sizes of formulated NNPs ranged from 199 ± 3.0 to 206 ± 7.7 nm for NNP1 and NNP3, respectively and from 215 ± 4.1 to 253 ± 8.7 nm for NP1 and NP3, respectively. It is clear that while keeping the concentration of surfactant constant, increasing PLGA concentration resulted in increased particle size. This could be ascribed to increasing polymer concentration in turn increasing the viscosity of the organic phase, which increases the forces that resist particle breakdown, leading to larger NPs (Lucia et al., 2015). In addition, the increase in particle size could be caused by increasing viscosity of the dispersed phase, the polymer solution, resulting in poorer dispersibility of the PLGA solution into the aqueous phase (Dos et al., 2012).

#### 3.2.7. Effect of drug: polymer concentration on EE

EE values were similar for NPs formulated with BRU: PLGA 1:2 and 1:3. Values for NNPs ranged from 69.1 ± 1.2% to 39.1 ± 1.9% (BRU: PLGA 1:2) and 70.6 ± 0.9% to 40.1 ± 1.7% (BRU:PLGA 1:3), while those for PEG NPs ranged between 71.7 ± 1.4% and 37.5 ± 1.8% (BRU:PLGA 1:2) and between 73.2 ± 1.9% and 39.1 ± 1.5% (BRU: PLGA 1:3). However, EE for NPs formulated with BRU: PLGA 1:4 increased notably; values for NNPs ranged between 74.0 ± 2.5% and 41.9 ± 1.4%, while those of their PEG counterparts ranged between 77 ± 1.3% and 41.1 ± 1.1%. From these results, it is obvious that increasing PLGA concentration will increase the EE of both naked and PEG NPs; it is also evident that surface coating with PEG did not affect the EE of the drug. This could be ascribed to the fact that increasing the polymer concentration would probably increase the viscosity of the organic phase, thus, increasing the diffusional resistance between organic and aqueous phases, thereby entrapping more drug in the NPs (Nazimuddin et al., 2019). From these results, it is evident that the optimal BRU: PLGA ratio is 1:4 as that gives the greatest particle size and EE. These results are in accordance with Budhian et al. (2007), who found that increasing polymer concentration leads to a gradual increase in NP diameter and the EE of drug.

### 3.3. Quantitative determination of serum protein adsorption onto NPs surface

**Figure 4.**
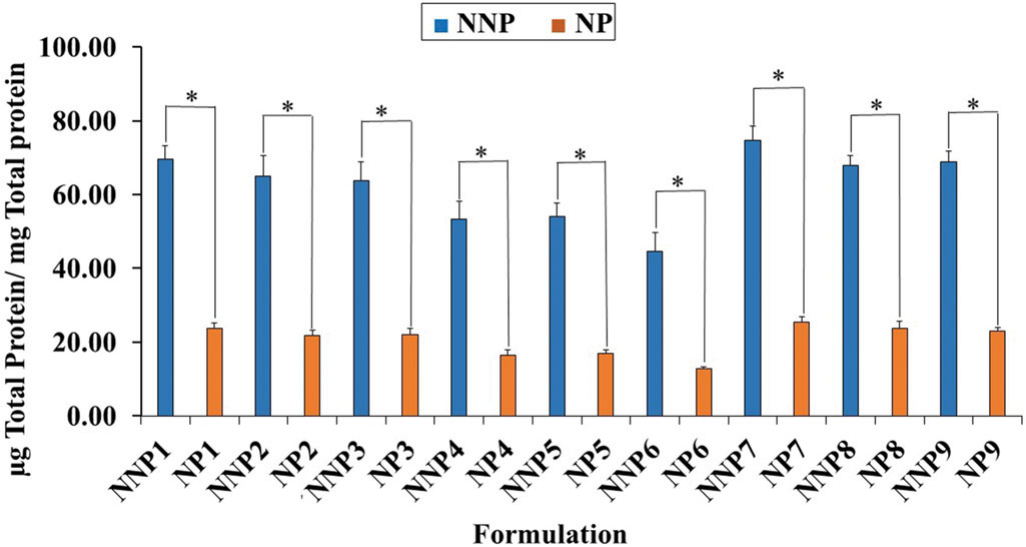
Total amount of serum proteins associated on the surface of naked and PEG PLGA NPs prepared with PVA. Results are expressed as the mean with the bar showing S.D. of three experiments. *p* < .05, compared with naked counterpart.

As shown in Figure 4, the total serum protein adsorbed on the surface of PEG NPs was significantly smaller than that on their naked counterparts. The quantity of adsorbed protein ranged from 14.9 ± 1.08 to 25.5 ± 1.5 µg/mg for PEG NPs (NP6 and NP7) and from 44.7 ± 5.0 to 74.7 ± 3.8 µg/mg for NNPs (NNP6 and NNP7). The lower adsorption of serum protein on PEG NPs could be ascribed to the presence of PEG on the surface of NPs (Shehata et al., 2008). This confirms the role of PEG in protecting NPs from recognition by RES, as it prevents serum proteins from recognizing and interacting with the NP surface (Shehata et al., 2016).

### 3.4. *In vitro* release of BRU from PLGA NPs

**Figure 5.**
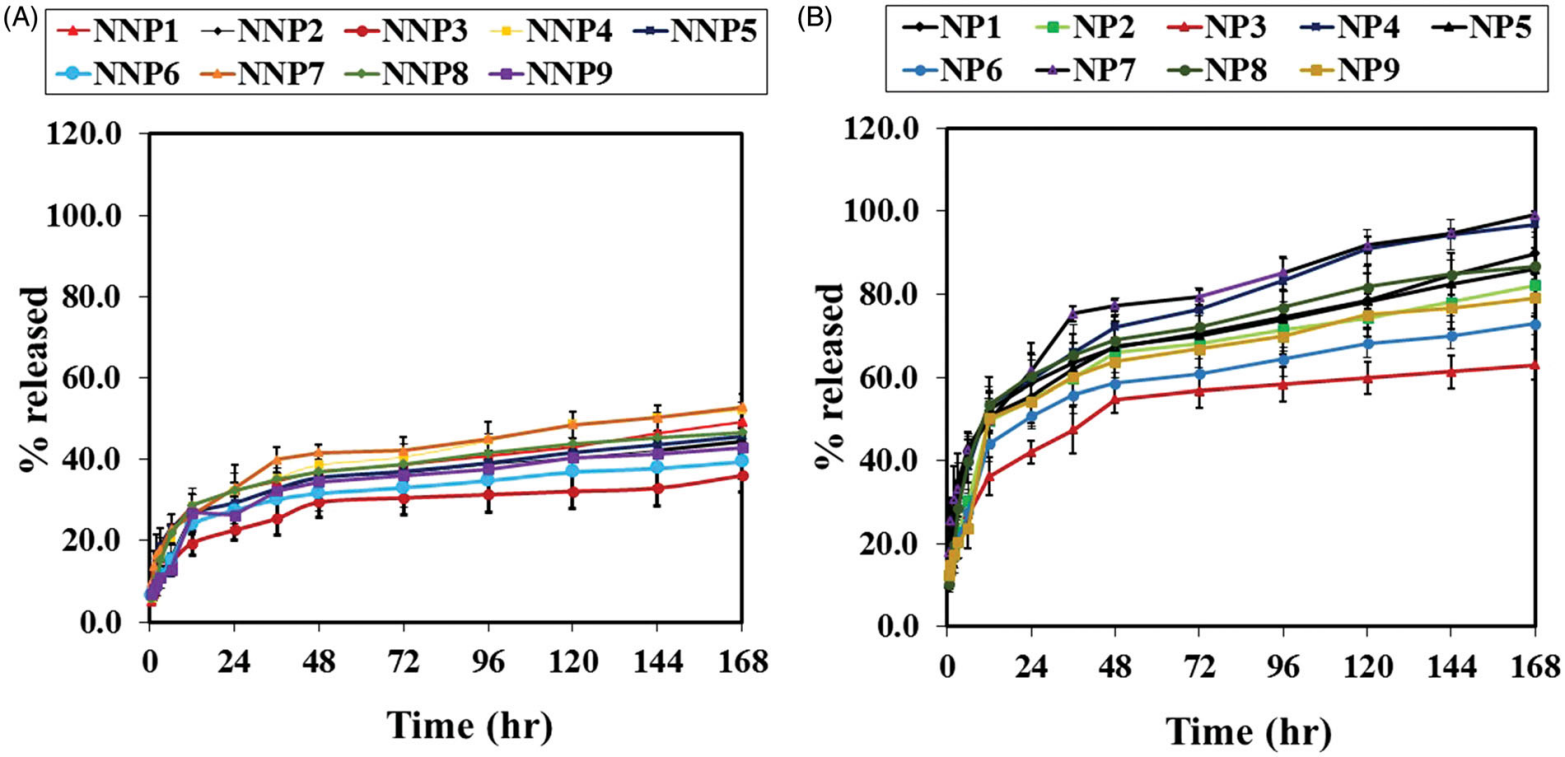
*In vitro* release studies of BRU A; from naked PLGA NPs prepared with PVA in PBS pH 7.4. B; from PEG PLGA NPs prepared with PVA in PBS pH 7.4. Results are expressed as the mean with the bar showing S.D. of three experiments. *p* < .05, compared with naked counterpart.

The *in vitro* release of BRU from PLGA NPS was profiled via a dialysis bag method that retained NPs and permitted diffusion of the drug into the receiving media. The release profiles of naked PLGA NPs are shown in Figure 5(A). After 168 h, the percentage of BRU released from NNPs (NNP1 to NNP9) ranged from 36 ± 4.2% to 52.8 ± 3.3%, with the lowest and highest being NNP3 and NNP7, respectively. Results from PEG NPs are given in Figure 5(B). After 168 h, the percentage of BRU released from PEG NPs (NP1 to NP9) ranged from 63 ± 3.7% to 99.1 ± 0.7%, with the lowest and highest being NP3 and NP7, respectively. Interestingly, PEG NPs showed faster and higher *in vitro* release than their naked counterparts. This could be attributed to the tendency of PEG molecules on the NP surface to attract water, leading to more wetting for PEG NPs and therefore higher drug release (Pedram & Azita, 2017). Another evident trend is that as the amount of PLGA increased, the percentage of drug released decreased. This could be attributed to the difference in particle size at different concentrations of PLGA, as NP size can affect the dissolution rate (Zili et al., 2005). Meanwhile, for a given PLGA concentration, increasing the amount of surfactant increased the percentage of BRU release. This behavior could also be explained on the basis of particle size: increasing surfactant concentration caused a decrease in NP size. Smaller NPs have more surface area relative to their volume, and hence a larger amount of drug is exposed and available to be released (Navneet et al., 2016).

### 3.5. Experimental design – 3^2^ level factorial design

Based on experimental design, following table (Table 6) showed result of particle size, % yield and protein adsorbed for different amount of PLGA and PVA.

**Table 6.** Experimental design results of particle size, % yield and protein adsorbed.

Batch no.	PLGA (mg)	PEG-DSPE (mg)	PVA (mg)	Particle size (nm ± SD)	Yield % ± SD	Protein adsorbed
NP01	85	50	15	217.4 ± 1.27	84.5 ± 1.58	22.4 ± 1.47
NP02	100	50	15	224.7 ± 2.01	89.2 ± 0.95	19.7 ± 1.25
NP03	115	50	15	257.2 ± 3.4	97.8 ± 1.02	18.4 ± 0.83
NP04	85	50	20	145.3 ± 1.0	81.5 ± 2.4	17.8 ± 0.84
NP05	100	50	20	161.4 ± 4.25	85.3 ± 3.25	14.9 ± 0.97
NP06	115	50	20	187.9 ± 2.5	89.7 ± 1.47	13.1 ± 1.14
NP07	85	50	25	93.7 ± 2.98	70.1 ± 2.11	24.8 ± 0.25
NP08	100	50	25	131.1 ± 3.01	76.8 ± 1.87	23.1 ± 1.47
NP09	115	50	25	158.4 ± 2.14	82.5 ± 1.3	22.7 ± 1.05
NP010	112	50	22	168.4 ± 3.85	83.5 ± 2.02	16.8 ± 0.52
NP011	95	50	18	174.5 ± 4.2	84.2 ± 0.98	15.2 ± 0.84

#### 3.5.1. Effect on particle size

**Figure 6.**
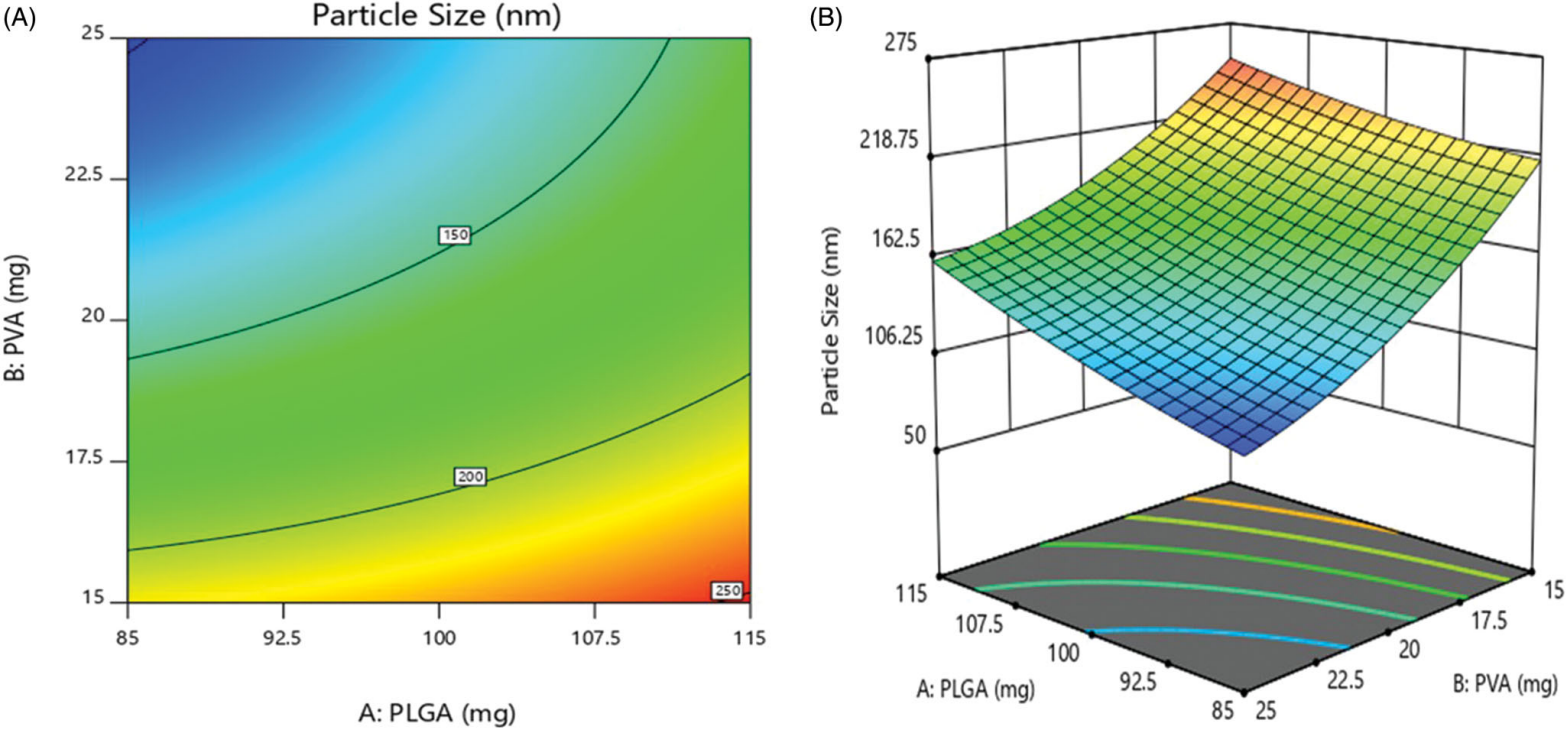
Effect of PEGylation on particle size (a) 2D – contour plot and (b) 3D – response surface plot.

The particle size was varied in range of 93.7 ± 2.8 nm to 257.2 ± 5.8 nm (Table 6). According to Figure 6(A,B), 2D contour plot and 3D-response surface plot showed that concentration of PLGA had non-significant effect on particle size while the significant inverse effect was observed with increase in the concentration of PVA surfactant. The data exhibited that the particle size was decreased as the concentration of PVA increased (Tefas et al., 2015; Vuddanda et al., 2015).

The regression coefficient for particle size was as follows:

$$Y1=161.7+24.51X1–52.68X2+6.225X1X2+4.{45X1}^{2}+15.{75X2}^{2}$$

The model was found significant with *F* value 91.95 (*p* = .0018), the coefficient of *r*^2^ was found to be 0.9935.

The viscosity of the organic phase was increased with increase in PLGA concentration. A higher viscosity leads to decrease shear stress and slow down the diffusion of organic phase into aqueous phase produces larger droplets which turn into render larger particle size (Song et al., 2008; Moacir et al., 2012). PVA can be occupied at the interface between the organic and aqueous phase, thus falling the interfacial tension and thereby increasing the shear stress. Therefore, this fact promotes the formation of small particle size. Further increase in PVA concentration, the viscosity of the aqueous phase increased, as a result decreased in the shear stress, and the mean diameter of particle size increased. Some results also indicate that higher PVA concentration endorses the coalescence of particles, leads to increase in particle size (Ravi et al., 2004; Mehrotra & Pandit, 2012; Tefas et al., 2015).

#### 3.5.2. Effect on % yield

**Figure 7.**
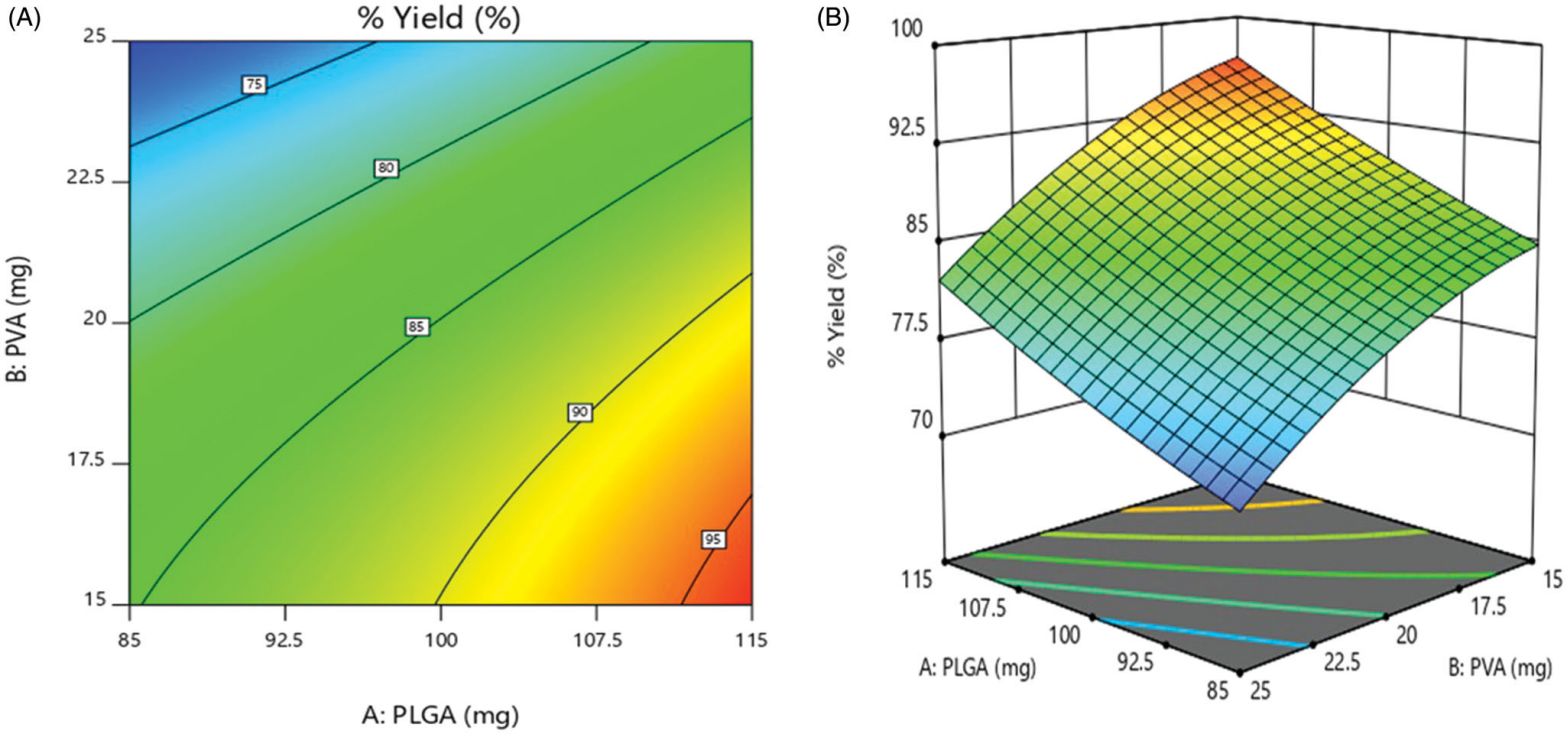
Effect on % yield (a) 2D – contour plot and (b) 3D – response surface plot.

For all the formulations, the % yield varied on a wide range from 70.1 ± 1.4% to 97.8 ± 1.1% (Table 6). As illustrated in Figure 7(A,B), 2D contour plot and 3D-response surface plot, a positive relationship was observed between % yield and concentration of PLGA. As the concentration of PLGA increased, % yield was increased. In contrast, the % yield was dramatically decreasing with increasing concentration of PVA (Vuddanda et al., 2015). The same relationship is observed in following equation.

The regression coefficient for % yield was as follows:

$$Y2=85.11+5.65X1-7.01–0.22X1X2+0.{58X1}^{2}–2.0{1X2}^{2}$$

The model was found significant with *F* value 32.04 (*p* = .0083), the coefficient of *r*^2^ was found to be 0.9816.

As mentioned above the viscosity of the aqueous solution was increased with the concentration of PVA, thereby reduction in shear stress. Thus a less favorable homogenization efficiency, low stirring rate and larger emulsion droplets, reduces the particle yield (Mehrotra & Pandit, 2012).

#### 3.5.3. Effect on protein adsorbed

**Figure 8.**
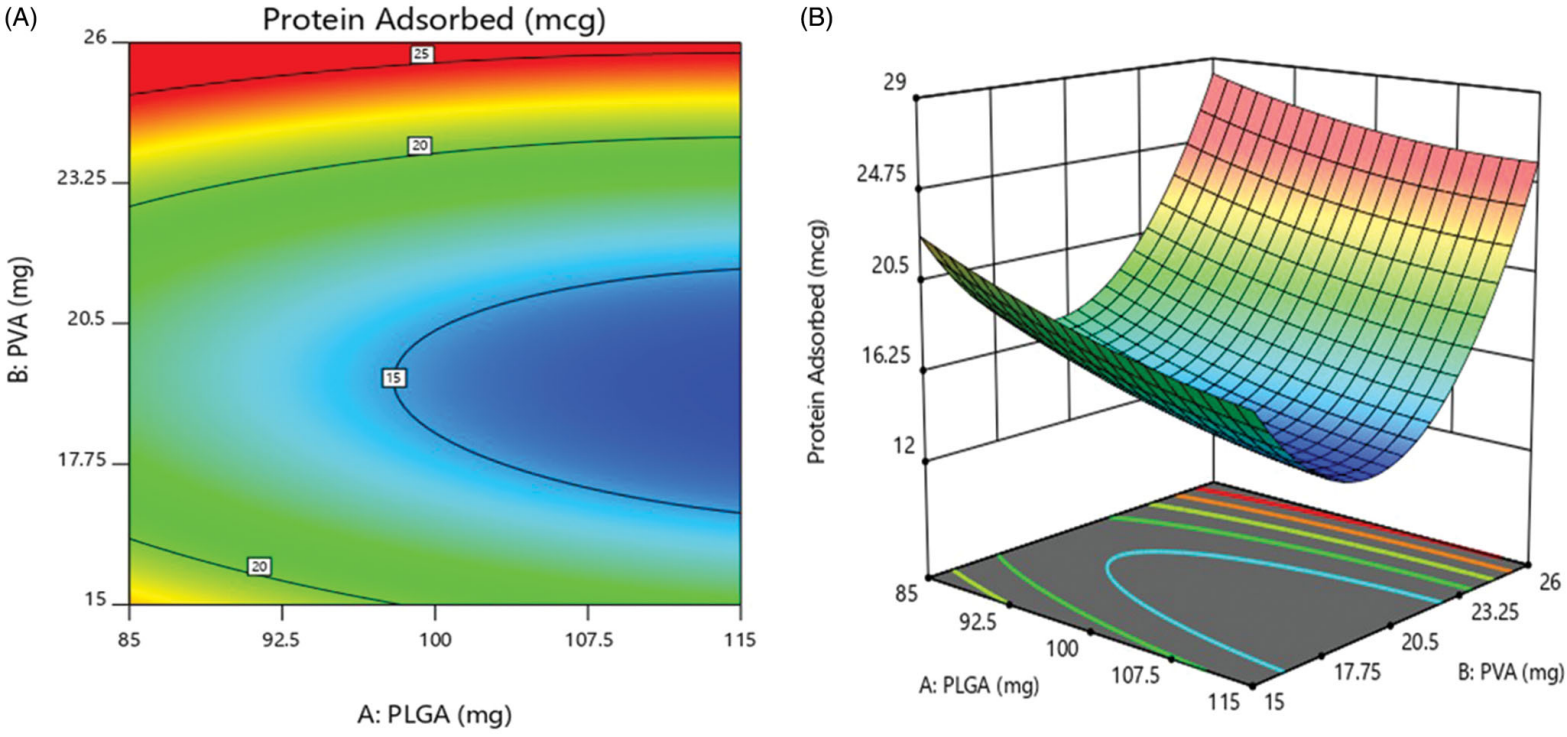
Effect on protein adsorbed (a) 2D – contour plot and (b) 3D – response surface plot.

The value of protein adsorbed for the designed formulations is in range of 13.1 ± 1.08% to 24.8 ± 2.5% as shown in Table 6. Figure 8(A,B) of 2D contour plot and 3D-response surface plot revealed the effect of the concentration of PEG presented on the surface of the nanoparticles. At the 20 mg concentration of PEG, minimum protein adsorption was observed. This could be due to effect of PEGylating inhibiting RES endocytosis of nanoparticles, as it prevents serum proteins from recognizing and interacting with the nanoparticles surface. After increasing concentration from 20 mg to 25 mg and decreasing concentration from 20 mg to 15 mg, the protein adsorption was increased. The figure showed that the concentration of PVA show disparate effect on the protein adsorption, as gradually increasing PVA concentration decreases protein adsorption to minimum initially and then further increased (Vuddanda et al., 2015).

The regression coefficient for protein adsorbed was as follows:

$$Y3=14.84–1.8X1+1.68X2+0.47X1X2+0.{63X1}^{2}+6.{58X2}^{2}$$

The model was found significant with *F* value 81.83 (*p* = .0021), the coefficient of *r*^2^ was found to be 0.9927.

Based on experimental design studies, the optimized batch NP05 was selected for further studies.

**Figure 9.**
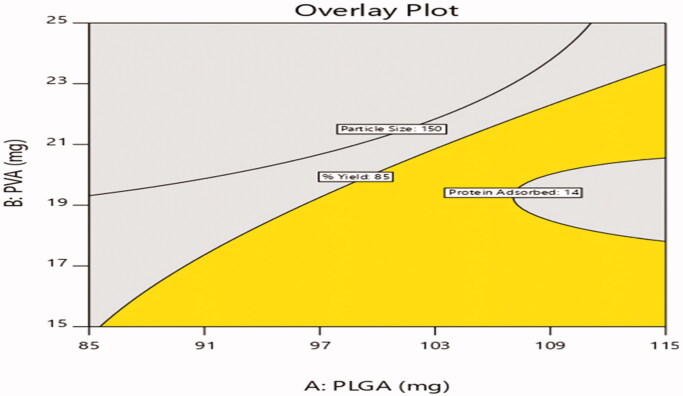
Design overlay plot.

The selected design was validated by selecting checkpoint batches based on overlay plot (Figure 9) and comparison of predicted values and observed values of dependent variables were shown in Table 7.

**Table 7.** Predicted and observed values of check point batches.

Batch no.	PLGA (mg)	PVA (mg)	Particle size		% Yield		Protein adsorbed	
			Predicted	Observed	Predicted	Observed	Predicted	Observed
NP010	112	22	167.7 ± 6.75	168.4 ± 3.85	86.8 ± 1.75	83.5 ± 2.02	15.68 ± 0.55	16.8 ± 0.52
NP011	95	18	178.4 ± 6.75	174.5 ± 4.2	85.7 ± 1.75	84.2 ± 0.98	15.95 ± 0.55	15.2 ± 0.84

### 3.6. In vivo anti-tumor activity of BRU-loaded NPs

**Figure 10.**
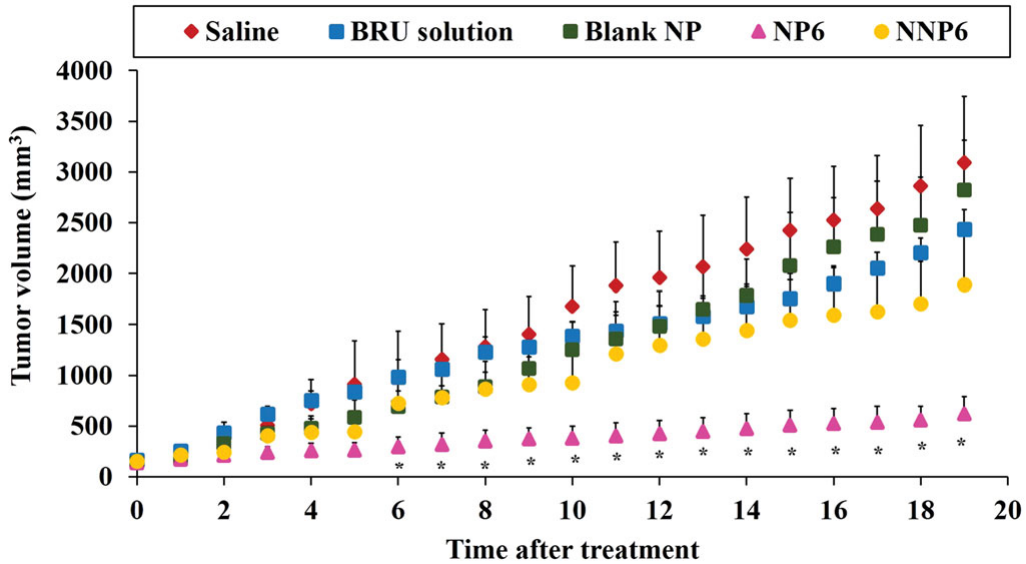
Effect of BRU-loaded PLGA NPs on tumor volume in MDA tumor bearing mice. * *p* < .05, compared with all groups under investigations.

**Figure 11.**
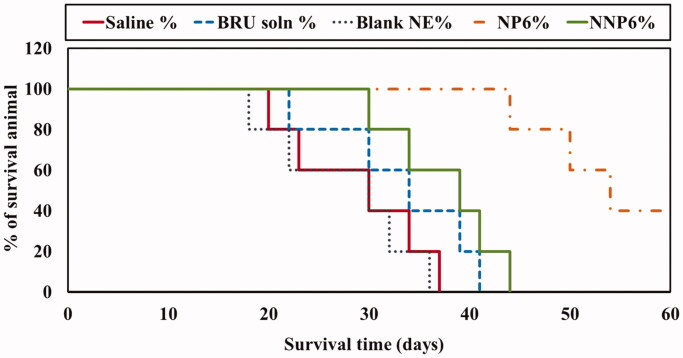
Effect of BRU-loaded PLGA NPs on survival of MDA tumor-bearing mice.

As described above, formulation NP6 (optimized formula NP05) had lower serum protein adsorption than the other preparations under investigation. It also showed suitable particle size, acceptable entrapment efficiency, good percent yield of the drug, low drug release after 168 h *in vitro*. Therefore, NP6 and its naked counterpart NNP6 were selected for the evaluation of *in vivo* antitumor activity in MDA tumor-bearing mice. Figure 10 illustrates changes in tumor volume following treatment, while Table 8 shows the tumor growth rates for preparations as calculated from the slope of the tumor volume-time curve. In addition, Figure 11 and Table 8 summarize the effects of BRU NPs on mice survival and the mean survival time (MST). The tumor volumes were 3093.2 ± 652.1, 2822.9 ± 490.4, 2432.5 ± 195.5, 1888.9 ± 525.1, and 619.6 ± 172.2 mm^3^ for groups treated with saline, blank NP, BRU solution, NNP6, and NP6, respectively (Figure 6). It is greatly evident that treatment with NP6 resulted in significantly smaller tumor volumes than any other treatment throughout the whole measuring period (*p* < .05). The tumor growth rates were 153.7 ± 43.6, 139.0 ± 34.5, 113.7 ± 13.0, 93.1 ± 30.1, and 23.11 ± 9.6 mm^3^/day for groups treated with saline, blank NP, BRU solution, NNP6, and NP6, respectively (Table 8). BRU solution alone had only a small effect on tumor growth, while tumor growth rates for the NP6 group were significantly lower than any other treatment (*p* < .05). in addition, Figure 10 shows that the tumor growth was not affected by blank NP treatment if compared with saline treated group (Xiao et al., 2017). The better effect of PEG NPs could be ascribed to their optimized particle size, which permits greater accumulation of BRU in tumor tissue through the EPR effect. When using Ecoflex® NPs loaded with docetaxel, Erfaneh et al. (2018) observed that excessive tumor growth in the control group was and remarkable inhibition in the treatment group. The present study also followed the survival of tumor-bearing mice for 60 days after treatment. Mean survival times were 28.4 ± 7.8, 27.6 ± 7.4, 33.2 ± 7.6, 38 ± 6.04, and 54.8 ± 7.4 days for groups treated with saline, blank NP, BRU solution, NNP6, and NP6, respectively (Table 8). No significant difference was found in survival between saline and blank NP treated groups (Xiao et al., 2017) while MST was significantly prolonged by treatment with PEG NPs (NP6) relative to all other treatment groups (*p* < .05). Similar results were observed by George et al. (2009), where treatment with cisplatin-loaded NP resulted in higher survival rates than with free cisplatin, blank NPs, or control treatment.

**Table 8.** Tumor growth rate and mean survival time (MST) values in tumor-bearing mice.

Parameters (unit)	Saline	Blank NP	BRU solution	NNP6	NP6
Tumor growth rate (mm^3^/day)	153.7 ± 43.6	139.0 ± 34.5	113.7 ± 13.0	93.1 ± 30.1	23.2 ± 9.5
			*	*	*
			**	**	**
					#
					■
Mean survival time (day)	28.8 ± 7.2	27.6 ± 7.4	33.2 ± 7.6	38 ± 6.04	54.8 ± 7 .4
				*	*
				**	**
					#
					■

Results are expressed as the mean ± S.D. of five mice.

* *p* < .05, compared with the saline group.

** *p* < .05, compared with the blank NP-treated group.

# *p* < .05, compared with the BRU solution-treated group.

■ *p* < 0.05, compared with the NNP6-treated group.

## 4. Conclusion

This study successfully developed BRU-loaded PLGA NPs using a modified solvent evaporation technique and confirmed that PLGA and surfactant concentration play major roles in determining NPs characteristics. The developed NPs proved to have appropriate particle sizes and suitable PDI for intravenous administration. Evaluation of plasma protein adsorption emphasized the role of PEG in reducing the amount of plasma protein on the NPs surface. *In vitro* release assays confirmed that BRU release can be successfully extended in PLGA NP formulations over a period of 186 h. Finally, evaluation of *in vivo* antitumor activity indicated that the developed PEGylated NPs can reduce tumor growth and prolong the survival time of MDA-bearing mice, which confirms the efficiency of BRU-loaded PEG PLGA NPs as a potential antitumor therapy.

## References

[CIT0001] Ahmed A, Saleh A, Shaaban O. (2015). Gold nanoparticles decorated with octreotide for somatostatin receptors targeting. J Pharm Sci Res 7:14–20.

[CIT0002] Budhian A, Siegel SJ, Winey KI. (2007). Haloperidol-loaded PLGA nanoparticles: systematic study of particle size and drug content. Int J Pharm 336:367–75. 17207944 10.1016/j.ijpharm.2006.11.061

[CIT0003] Catarina PR, Ronald JN, Antonio JR, Francisco V. (2006). Nanoencapsulation I. Methods for preparation of drug-loaded polymeric nanoparticles. Nanomed Nanotechnol Biol Med 2:8–21. 10.1016/j.nano.2005.12.003 17292111

[CIT0004] Danaei M, Dehghankhold M, Ataei S, et al. (2018). Impact of particle size and Polydispersity Index on the clinical applications of lipidic nanocarrier systems. Pharmaceutics 10:57–17. 10.3390/pharmaceutics10020057 PMC6027495 29783687

[CIT0005] Dos S, da S, Pereira-Filho E, et al. (2012). Polymeric nanoparticles loaded with the 3,5,3-triiodothyroacetic acid (Triac), a thyroid hormone: factorial design, characterization, and release kinetics. Nanotechnol Sci Appl 5:37–48. 24198495 10.2147/NSA.S32837 PMC3781720

[CIT0006] Erfaneh G, Jaleh V, Ali J, et al. (2018). Pharmacokinetics and in vitro/in vivo antitumor efficacy of aptamer-targeted Ecoflex® nanoparticles for docetaxel delivery in ovarian cancer. Int J Nanomed 2018:493–504. 10.2147/IJN.S152474 PMC5789074 29416331

[CIT0007] George M, Era T, Sylva H, et al. (2009). In vivo investigation of tolerance and antitumor activity of cisplatin-loaded PLGA-mPEG nanoparticles. Eur J Pharm Biopharm 71:190–5. 18929649 10.1016/j.ejpb.2008.09.011

[CIT0008] Govender J, Stolnik S, Garnett M, Illum L. (1999). PLGA nanoparticles prepared by nanoprecipitation: drug loading and release studies of a water soluble drug. J Control Release 57:171–85. 9971898 10.1016/s0168-3659(98)00116-3

[CIT0009] Gupta A, Chaphalkar S. (2015). Cytotoxic and anti-inflammatory activity of aqueous extract of Strychnosnux-vomica. J Biol Nat 4:217–23.

[CIT0010] Hoa L, Nguyen T, Le H, Dang M. (2012). Preparation and characterization of nanoparticles containing ketoprofen and acrylic polymers prepared by emulsion solvent evaporation method. J Exp Nanosci 7:189–97.

[CIT0011] Ismail R, Sovány T, Gácsi A, et al. (2019). Synthesis and statistical optimization of poly (lactic-*co*-glycolic acid) nanoparticles encapsulating GLP1 analog designed for oral delivery. Pharm Res 36:1–16. 10.1007/s11095-019-2620-9 PMC6513835 31087188

[CIT0012] Issa A, Mohammad R, Motahare-Sadat H. (2013). Anticancer activity of nanoparticles based on PLGA and its co-polymer: in-vitro evaluation. Iran J Pharm Res 12:623–34. 24523742 PMC3920687

[CIT0013] Jiang W, Kim B, Rutka J, Chan W. (2007). Advances and challenges of nanotechnology-based drug delivery systems. Expert Opin Drug Deliv 4:621–33. 17970665 10.1517/17425247.4.6.621

[CIT0014] Jörg K, Telli H, Sebastian D, et al. (2007). Covalent attachment of apolipoprotein A-I and apolipoprotein B-100 to albumin nanoparticles enables drug transport into the brain. J Control Release 118:54–8. 17250920 10.1016/j.jconrel.2006.12.012

[CIT0015] Keum c, Young-Wook N, Jong-suep B, et al. (2011). Practical preparation procedures for docetaxel-loaded nanoparticles using polylactic acid-co-glycolic acid. Int J Nanomedicine 6:2225–34. 22114486 10.2147/IJN.S24547 PMC3215163

[CIT0016] Kozaki M, Kobayashi SI, Goda Y, et al. (2017). Evaluating the properties of poly(lactic-co-glycolic acid) nanoparticle formulations encapsulating a hydrophobic drug by using the quality by design approach . Chem Pharm Bull 65:218–28. 10.1248/cpb.c16-00415 28250343

[CIT0017] Lee E, Na K, Bae Y. (2005). Doxorubicin loaded pH-sensitive polymeric micelles for reversal of resistant MCF-7 tumor. J Control Release 103:405–18. 15763623 10.1016/j.jconrel.2004.12.018

[CIT0018] Lövestam G, Rauscher H, Roebben G, et al. (2010). Considerations on a definition of nanomaterial for regulatory purposes. Luxembourg: Publications Office of the European Union.

[CIT0019] Lucia R, Ioan T, Marcela A, Laurian V. (2015). Development and optimization of quercetin loaded PLGA nanoparticles by experimental design. Clujul Medical 88:214–23. 26528074 10.15386/cjmed-418 PMC4576773

[CIT0020] Mehrotra A, Pandit JK. (2012). Critical process parameters evaluation of modified nanoprecipitation method on lomustine nanoparticles and cytostatic activity study on L132 human cancer cell line. J Nanomed Nanotechnol 3:6.

[CIT0021] Moacir RF, SKC d, da SM, et al. (2012). Polymeric nanoparticles loaded with the 3,5,3-triiodothyroacetic acid (Triac), a thyroid hormone: factorial design, characterization, and release kinetics. Nanotechnol Sci Appl 2012:37–48. 10.2147/NSA.S32837 PMC3781720 24198495

[CIT0022] Mohamed F, Nicolas A, Justine W, et al. (2019). An overview of active and passive targeting strategies to improve the nanocarriers efficiency to tumour sites. J Pharm Pharmacol 71:1185–1198. 31049986 10.1111/jphp.13098

[CIT0023] Mohammed H, Urszula D. (2014). PLGA biodegradable nanoparticles containing perphenazine or chlorpromazine hydrochloride: effect of formulation and release. Int J Mol Sci 15:23909–23. 25535080 10.3390/ijms151223909 PMC4284797

[CIT0024] Morsy MA, Abdel-Latif RG, Nair AB, et al. (2019). Preparation and evaluation of atorvastatin-loaded nanoemulgel on wound-healing efficacy. Pharmaceutics 11:609. 10.3390/pharmaceutics11110609 PMC6920749 31766305

[CIT0025] Navneet S, Parshotam M, Senshang L. (2016). Effect of process and formulation variables on the preparation of parenteral paclitaxel-loaded biodegradable polymeric nanoparticles: a co-surfactant study. Asian J Pharm Sci 11:404–16.

[CIT0026] Nazimuddin C, Satveer J, Dinesh D, et al. (2019). Preparation, optimization, and in vivo evaluation of nanoparticle-based formulation for pulmonary delivery of anticancer drug. Medicina 55: 294. 10.3390/medicina55060294 PMC6631245 31226865

[CIT0027] Ogawara K, Un K, Minato K, et al. (2008). Determinants for in vivo anti-tumor effects of PEG liposomal doxorubicin: importance of vascular permeability within tumors. Int J Pharm 359:234–40. 18448289 10.1016/j.ijpharm.2008.03.025

[CIT0028] Patel B, Gupta V, Ahsan F. (2012). PEG-PLGA based large porous particles for pulmonary delivery of a highly soluble drug, low molecular weight heparin. J Control Release 162:310–20. 22800582 10.1016/j.jconrel.2012.07.003

[CIT0029] Pedram R, Azita H. (2017). Docetaxel-loaded PLGA and PLGA-PEG nanoparticles for intravenous application: pharmacokinetics and bio distribution profile. Int J Nanomed 12:935–47. 10.2147/IJN.S121881 PMC5291330 28184163

[CIT0030] Peng Y, Nie J, Cheng W, et al. (2018). A multifunctional nanoplatform for cancer chemo-photothermal synergistic therapy and overcoming multidrug resistance. Biomater Sci 6:1084–98. 29512657 10.1039/c7bm01206c

[CIT0031] Prakash C, Amita V, Fahad A, et al. (2017). Development of surface-engineered PLGA nanoparticulate-delivery system of Tet1-conjugated nattokinase enzyme for inhibition of Aβ40 plaques in Alzheimer’s disease. Int J Nanomed 12:8749–68. 10.2147/IJN.S144545 PMC5732557 29263666

[CIT0032] Qin J, Pei-Hao Y, Qi L, et al. (2012). Anti-tumor effects of brucine immuno-nanoparticles on hepatocellular carcinoma. Int J Nanomed 7:369–79. 10.2147/IJN.S27226 PMC3273973 22334771

[CIT0033] Rao JP, Geckeler KE. (2011). Polymer nanoparticles: preparation techniques and size-control parameters. Prog Polym Sci 36:887–913.

[CIT0034] Ravi K, Bakowsky U, Lehr CM. (2004). Preparation and characterization of cationic PLGA nanospheres as DNA carriers. Biomaterials 25:1771–7. 14738840 10.1016/j.biomaterials.2003.08.069

[CIT0035] Rekha K, Jyoti S, Vinay S. (2014). Development and characterization of nanoparticles for the delivery of gemcitabine hydrochloride. Sci World J 2014:1–6. 10.1155/2014/560962 PMC3925564 24592173

[CIT0036] Rizwan K, Muhammad A, Sarfaraz K, et al. (2019). The influence of ionic and nonionic surfactants on the colloidal stability and removal of CuO nanoparticles from water by chemical coagulation. Int J Environ Res Public Health 16:1–17. 10.3390/ijerph16071260 PMC6479800 30970550

[CIT0037] Rodríguez A, Batlle R, Nerín C. (2007). The use of natural essential oils as antimicrobial solutions in paper packaging. Part II. Prog Org Coat 60:33–8.

[CIT0038] Rubiana M, Maria P, Raul C. (2006). Thermoanalytical study of praziquantel-loaded PLGA nanoparticles. Braz J Pharm Sci 42:523–30.

[CIT0039] Sempf K, Tabiwang A, Svetlana G, et al. (2013). Adsorption of plasma proteins on uncoated PLGA nanoparticles. Eur J Pharm Biopharm 85:53–60. 23395970 10.1016/j.ejpb.2012.11.030

[CIT0040] Shah J, Nair AB, Jacob S, et al. (2019). Nanoemulsion based vehicle for effective ocular delivery of moxifloxacin using experimental design and pharmacokinetic study in rabbits. Pharmaceutics 11:230. 10.3390/pharmaceutics11050230 PMC6571706 31083593

[CIT0041] Shehata T, Ken-ichi O, Kazutaka H, Toshikiro K. (2008). Prolongation of residence time of liposome by surface-modification with mixture of hydrophilic polymers. Int J Pharm 359:272–9. 18486370 10.1016/j.ijpharm.2008.04.004

[CIT0042] Shehata T, Toshikiro K, Kazutaka H, Ken-ichi O. (2016). In-vivo disposition characteristics of PEG niosome and its interaction with serum proteins. Int J Pharm 512:322–8. 27586409 10.1016/j.ijpharm.2016.08.058

[CIT0043] Shu L, Xi-Peng W. (2017). In vitro and in vivo evaluation of novel NGR-modified liposomes containing brucine. IJN 12:5797–804. 28860749 10.2147/IJN.S136378 PMC5565249

[CIT0044] Siqi Y, Linzhu Z, Yue S, et al. (2019). One-pot photoreduction to prepare NIR-absorbing plasmonic gold nanoparticles tethered by amphiphilic polypeptide copolymer for synergistic photothermal-chemotherapy. Chin Chem Lett 30:187–91.

[CIT0045] Song X, Zhao Y, Hou S, et al. (2008). Dual agents loaded PLGA nanoparticles: systematic study of particle size and drug entrapment efficiency. Eur J Pharm Biopharm 69:445–53. 18374554 10.1016/j.ejpb.2008.01.013

[CIT0046] Sovan LP, Utpal J, Manna PK, et al. (2011). Nanoparticle: an overview of preparation and characterization. J Appl Pharm Sci 01:228–34.

[CIT0047] Tefas LR, Tomuţă I, Achim M, Vlase L. (2015). Development and optimization of quercetin-loaded plga nanoparticles by experimental design. Clujul Med 88:214–23. 26528074 10.15386/cjmed-418 PMC4576773

[CIT0048] Venkatasubbu G, Ramasamy S, Avadhani G, et al. (2013). Surface modification and paclitaxel drug delivery of folic acid modified polyethylene glycol functionalized hydroxyapatite nanoparticles. Powder Technol 235:437–42.

[CIT0049] Vuddanda PR, Mishra A, Singh SK, Singh S. (2015). Development of polymeric nanoparticles with highly entrapped herbal hydrophilic drug using nanoprecipitation technique: an approach of quality by design. Pharm Dev Technol 20:579–87. 24831535 10.3109/10837450.2014.908302

[CIT0050] Wang Y, Puwang L, Lingxue K. (2013). Chitosan-modified PLGA nanoparticles with versatile surface for improved drug delivery. AAPS PharmSciTech 14:585–92. 23463262 10.1208/s12249-013-9943-3 PMC3665987

[CIT0051] Xiao LN, Long XC, Heng Z, et al. (2017). In vitro and in vivo antitumor effect of gefitinib nanoparticles on human lung cancer. Drug Deliv 24:1501–12. 28961023 10.1080/10717544.2017.1384862 PMC8241075

[CIT0052] Xiaowei Z, Wei T, Zhongyuan W, et al. (2015). Docetaxel-loaded nanoparticles of dendritic amphiphilic block copolymer H40-PLA- b -TPGS for cancer treatment. Part Part Syst Charact 32:112–22.

[CIT0053] Yang R, Han X, Shia X, et al. (2009). Cationic formulation of paclitaxel-loaded poly D, L-lactic-co-glycolic acid (PLGA) nanoparticles using an emulsion-solvent diffusion method. Asian J Pharm Sci 4:89–95.

[CIT0054] Yuan B, Mingjiang Y, Xiao W, et al. (2018). Antitumor activity of arsenite in combination with tetrandrine against human breast cancer cell line MDA-MB-231 in vitro and in vivo. Cancer Cell Int 18:113–4. 30123091 10.1186/s12935-018-0613-0 PMC6090820

[CIT0055] Zhipeng Chen J, Wu L, Li W, et al. (2013). Hyaluronic acid-coated bovine serum albumin nanoparticles loaded with brucine as selective nanovectors for intra-articular injection. Int J Nanomed 8:3843–53. 10.2147/IJN.S50721 PMC3794990 24124369

[CIT0056] Zili Z, Sfar S, Fessi H. (2005). Preparation and characterization of poly-epsilon-caprolactone nanoparticles containing griseofulvin . Int J Pharm 294:261–7. 15814249 10.1016/j.ijpharm.2005.01.020

